# Aurophilic Molecules on Surfaces. Part II. (NapNC)AuCl on Au(111)

**DOI:** 10.1021/acsomega.3c04152

**Published:** 2023-10-06

**Authors:** Thorsten Wagner, Michael Györök, Sebastian Wolfmayr, Petra Gründlinger, Uwe Monkowius, Peter Zeppenfeld

**Affiliations:** † Institute of Experimental Physics, Surface Science Division, Johannes Kepler University, Altenberger Straße 69, 4040 Linz, Austria; ‡ School of Education, Chemistry, Johannes Kepler University, Altenberger Straße 69, 4040 Linz, Austria

## Abstract

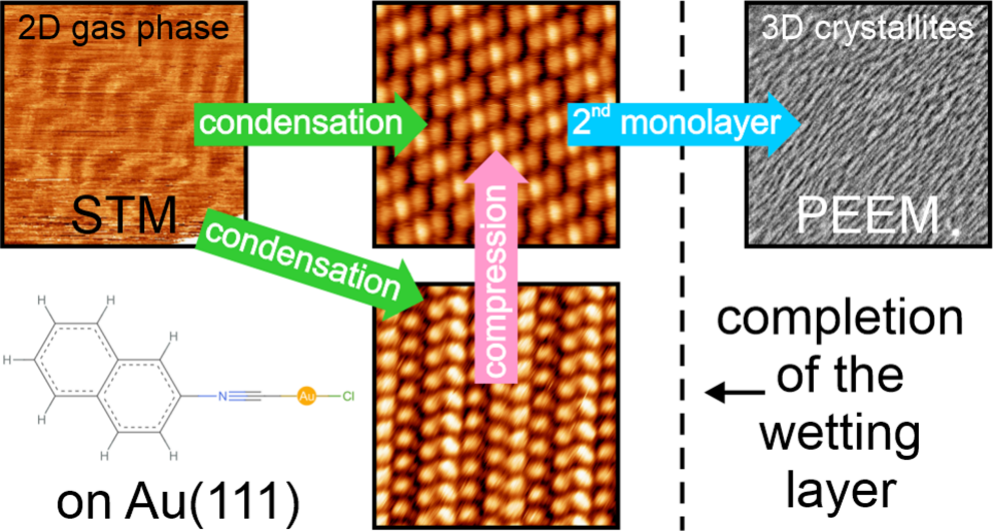

Although aurophilicity is a well-known phenomenon in structural gold chemistry and is found in many crystals of Au(I) complexes, its potential for self-assembly in thin films is not yet explored. This paper is Part II of a study, in which we investigated the ultrathin film formation of chlorido(2-naphthyl isonitrile) gold(I) on gold surfaces. Here, we present the data for the growth of (NapNC)AuCl on isotropic Au(111) surfaces. Already during physical vapor deposition, the condensation of ultrathin films is monitored by photoelectron emission microscopy (PEEM) and incremental and spectrally resolved changes in the optical reflectance (DDRS). Additional structural data obtained by scanning tunneling microscopy (STM) and low-energy electron diffraction (LEED) reveal that the “crossed swords” packing motif known from the bulk is also present in thin films.

## 1 Introduction

Well-ordered structures on surfaces, e.g., three-dimensional (3D) crystals or two-dimensional (2D) films, are often the key to access material properties of the constituting molecules.^1^ If there is only a short-range order, the material properties are strongly affected by defects acting, for example, as charge traps.^2^ However, well-defined attractive interactions between complex organic molecules are a prerequisite for them to form well-ordered condensed structures on surfaces. Although van der Waals forces are omnipresent and very often the driving force, they are neither directed nor very specific: usually, the extended π-electron systems are responsible for high binding energies between aromatic molecules and/or between the molecules and the substrate surface. Often this nonspecific interaction leads to a zoo of polymorphs not only in the bulk phase but also, in particular, in ultrathin films. Therefore, it is tempting to achieve a more localized interaction between the molecules. Aurophilic attraction may play this role.^3,4^ Relativistic effects in Au(I) complexes lead to an effective attractive interaction confined to the metal ion.^5,6^ This phenomenon is not restricted to gold but was also observed for other coinage metals. However, due to their high atomic mass, it is strongest for gold atoms.^7^

**Figure 1:**
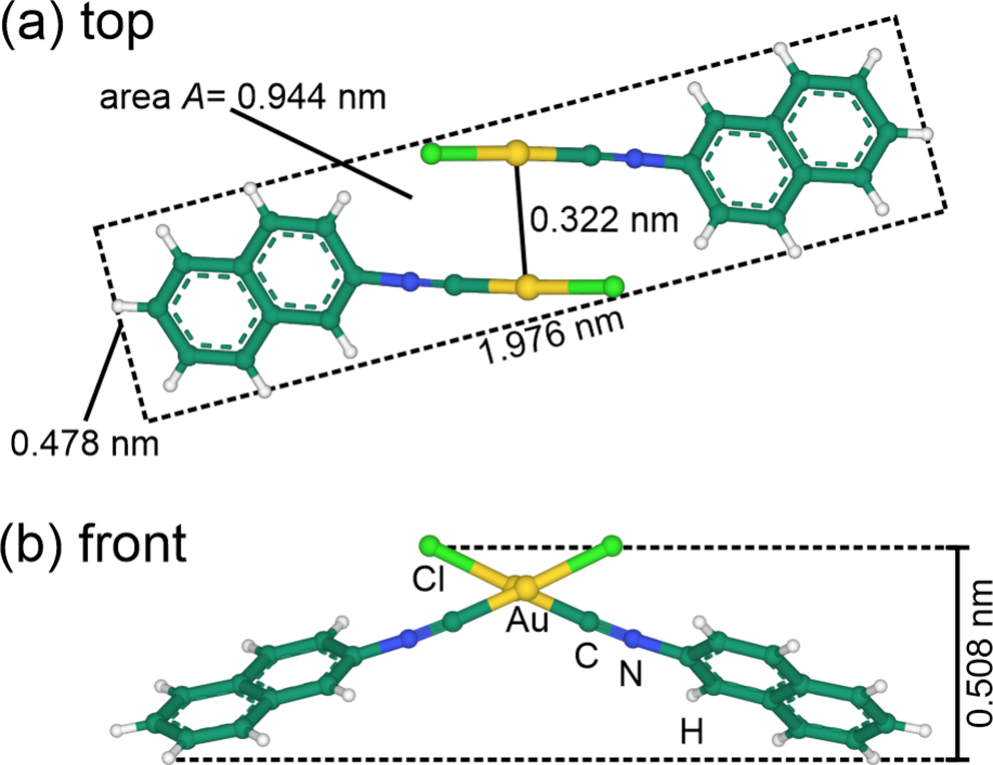
(a) Top view of a “crossed swords” dimer. The dashed rectangle indicates the smallest possible rectangular unit cell in this projection. The size of the rectangle is defined only by the positions of the atoms; the respective van der Waals radii are not considered. (b) View from the front onto the same configuration. The vertical distance between the lowest hydrogen atoms and the chlorine atoms is marked. The structural data are taken from ref (10). Mol* was used for visualization.^11^

In two consecutive papers, we report on the interaction of chlorido(2-naphthyl isonitrile)gold(I) (see Figure 1) on and with gold surfaces.^8^ Note that we will use in the following (NapNC)AuCl as the abbreviation to be consistent with previous publications (see refs (9) and (10)) and to illustrate the connectivity, although the IUPAC standard suggests [AuCl(NapNC)]. In the bulk phase, (NapNC)AuCl forms so-called “crossed swords” dimers via aurophilic bonding as reported by Hobbollahi and co-workers.^10^ We also proved the thermal stability of (NapNC)AuCl upon physical vapor deposition on glassy carbon.^9^ In the first part of this study, we reported on the growth of (NapNC)AuCl on anisotropic Au(110) surfaces.^8^ In this second part, we discuss our experiments on isotropic Au(111) surfaces. The measurement methodology was identical in both parts of the work in order to ensure the comparability of the results.

**Figure 2:**
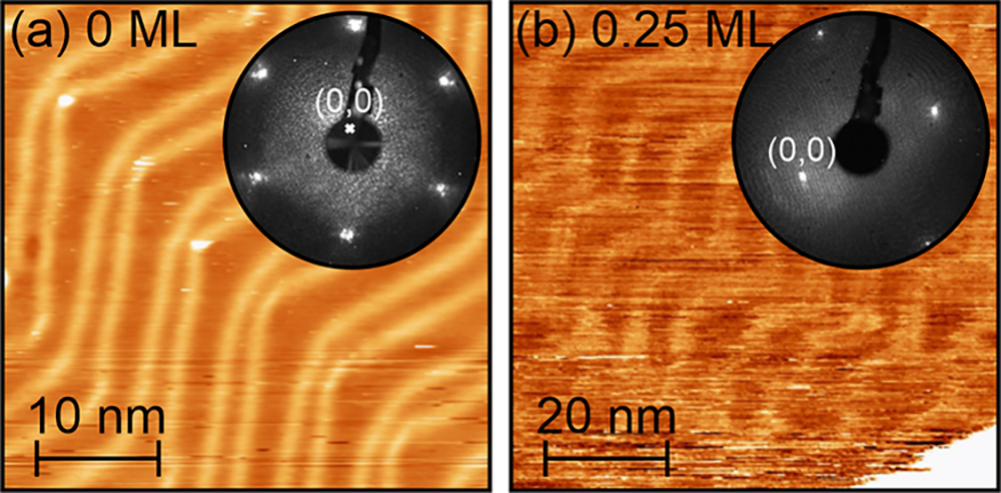
(a) STM image of a reconstructed Au(111)(22 × ) surface. The image size is 40 × 40 nm^2^. The image was acquired in constant current mode with *U*_sample_ = 700 mV and *I*_T_ = 1 nA. The low-energy electron diffraction (LEED) pattern shown as an inset was recorded with an electron energy of 66.8 eV. (b) STM image and LEED pattern after deposition of about 0.25 ML of (NapNC)AuCl and subsequent annealing to 363 K for 5 min. The STM image (Fourier-filtered to remove an instrumental artifact) shows an area of 80 × 80 nm^2^ recorded with *U*_sample_ = 50 mV and *I*_T_ = 0.03 nA. The LEED pattern was acquired with an electron energy of 40.6 eV in off-axis geometry to make the halo around the (0,0) spot visible. Both STM image use an almost identical *z*-scale.

After careful preparation of a Au(111) single crystal in vacuum, usually a characteristic herringbone reconstruction is found: along the ⟨101̅⟩ direction, 23 atoms of the first layer span 22 lattice sites of the bulk structure.^12,13^ The stacking changes from fcc (faced centered cubic), which is characteristic for the bulk, to hcp (hexagonal closed packed) stacking. In between these fcc and hcp domains, the atoms of the first layer occupy bridge sites, which are Δ*h* = 0.014 nm higher than the hollow sites of the fcc and hcp regions. Therefore, the characteristic domain boundaries (also called “soliton walls”) can be imaged by scanning tunneling microscopy (STM) as linear protrusions (see Figure 2a). In the literature, this packing motif is often referred to as ‘(22 × ) reconstruction’. Actually, the implicitly used Wood notation is not able to describe this rectangular structure, which in the matrix notation should read as follows: .

The characteristic “zigzag” pattern arises, when the direction of the soliton walls changes (every 10–20 nm) by 120° resulting in the typical “herringbone pattern”.^14^ The reconstruction gives rise to additional spots around the low-order LEED spots (see Figure 2a). The exact reconstruction is also affected by strain.^15,16^ If small amounts of adsorbates (atoms or molecules) are deposited on such a reconstructed Au(111) surface, they often adsorb preferentially at the elbows.^14,17^ For higher coverages, the herringbone reconstruction may also be lifted.^18,19^

## 2 Experimental Details

All experiments were carried out in situ in an ultrahigh vacuum system with a base pressure below 5 × 10^–10^ mbar. The vacuum vessel hosts a Focus PEEM with an integrated sample stage, an Omicron SpectaLEED, and an Omicron VT-AFM (operated as STM in constant current mode). Before each thin film preparation, the surface was prepared by repeated cycles of sputtering with Ar ions for about 30 min (Ar^+^ energy of 900 eV, current density of ≈2.5 μA cm^–2^, angle of incidence of ≈45°) and subsequent annealing at 850 K for 5 min. The presence of the herringbone reconstruction and, therefore, the cleanliness of the surface were confirmed by LEED and STM (see Figure 2a). Besides the diffraction spots of the bulk, also satellite spots around the higher-order spots are visible confirming the presence of the herringbone reconstruction.

**Figure 3:**
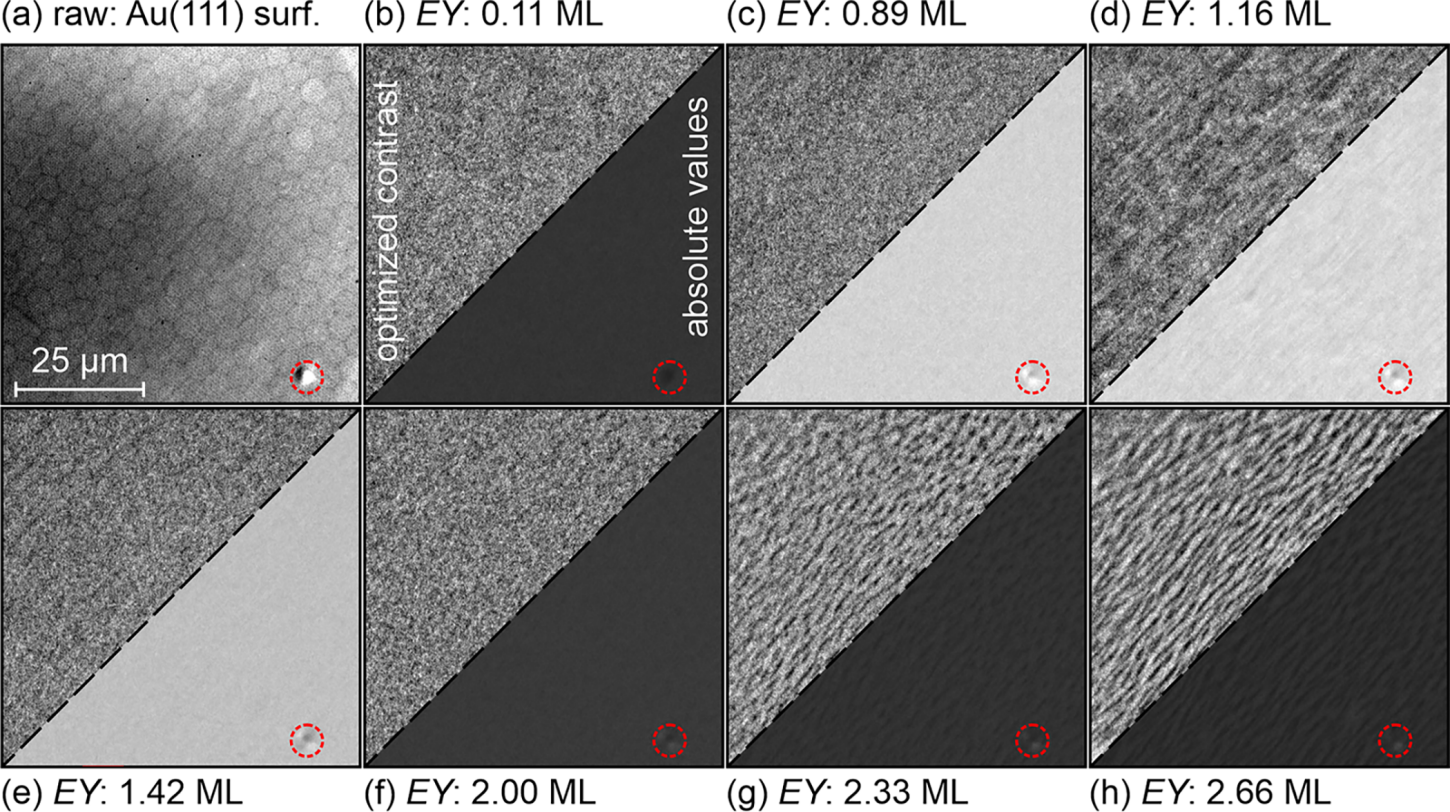
PEEM images with a size of 70 μm × 70 μm. (a) Average of 75 images before opening the shutter of the evaporator. (b–h) The images were normalized according to eq 1 representing the local variation of the electron yield at given coverages. The lower right corner of the images represents all images with the same settings of the gray scale allowing one to follow changes in the local electron yield. The upper left corner shows the region of interest with individually optimized contrast to make small variations better visible. The red dashed circles mark a defect (dust particle) already present on the bare surface.

The (NapNC)AuCl films were prepared by thermal evaporation (OVD3 from ventiotec). The quartz crucible was kept at a constant temperature of 403.15 K by using a PID controller. In a previous study, we showed that (NapNC)AuCl sublimates without decomposition at this temperature.^9^ During the deposition of (NapNC)AuCl onto the reconstructed Au(111) surface, PEEM images were continuously acquired with a frame rate of one image per second. The substrate was at room temperature during the deposition process and the subsequent characterization with LEED and STM. For the excitation of the photoelectrons, a super quiet Xe lamp (Hamamatsu) was used. Selected images of such a deposition experiment are shown in Figure 3. An average of 75 raw images recorded before opening the shutter (*B*(*x*, *y*)), from which a dark image (*D*(*x*, *y*)) was subtracted, is shown in Figure 3a. This dark image was obtained by averaging a series of images acquired without illumination. The raw image of the clean surface, *B*(*x*, *y*), shows a defect or dust particle (red circle) on the surface and a number of instrumental artifacts related to the imaging system such as the hexagonal structure of the microchannel plates (MCPs). All consecutive images recorded during (NapNC)AuCl deposition were processed according to
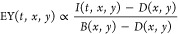
1where EY(*t*, *x*, *y*) denotes the electron yield and *I*(*t*, *x*, *y*) denotes the measured image intensity at time *t* and pixel coordinates (*x*, *y*). After subtraction of the dark image, *D*(*x*, *y*), the result is normalized by *B*(*x*, *y*) – *D*(*x*, *y*). In this way, the instrumental artifacts but also the initial inhomogeneity of the bare surface are effectively removed.

**Figure 4:**
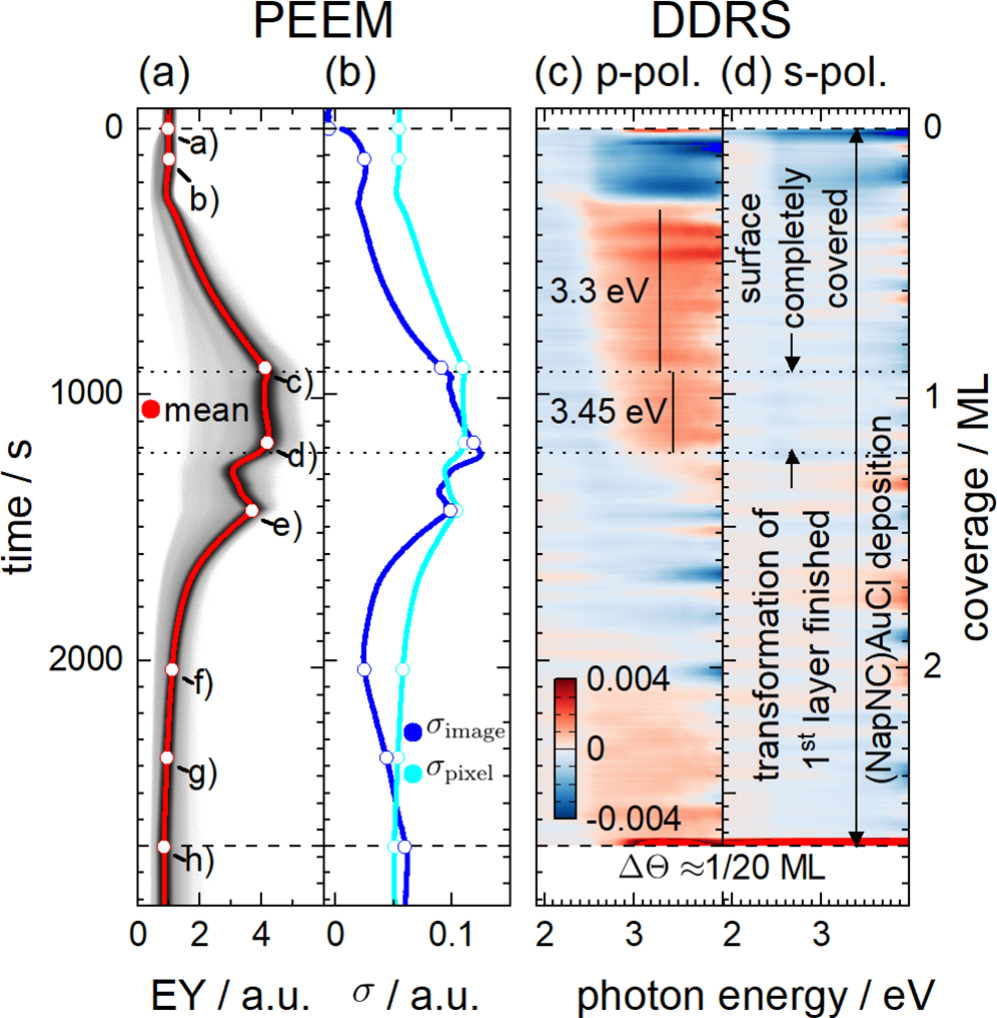
(a) Distribution of the electron yield (EY) of individual images shown as rows in a nonlinear false color representation of the respective histograms. The mean electron yield (MEY) is plotted as a red line. (b) Standard deviation related to the pixel noise (σ_pixel_) and the inhomogeneity of the images due to the morphology (σ_image_). The open circles in panels (a, b) mark the data points related to the images shown in Figure 3. The DDRS signal is calculated according to eq 2 for (c) p- and (d) s-polarized light. In all graphs, the left axis represents the deposition time (after opening the shutter). The right axis represents the corresponding coverages, assuming a constant deposition rate.

As described in ref (20), the changes in the optical reflectivity can be measured for s- and p-polarized light (pol-DRS) simultaneously and synchronized to the acquisition of the PEEM images. Since the electronic properties of ultrathin films change, and with them the reflectivity of the sample, the deposition process can be monitored in real time by DRS.^21,22^ Using polarized light, it is even possible to follow the 3D orientation of the transition dipole moment and thus the possible reorientation of elongated or asymmetric molecules.^23^ For PEEM imaging and differential reflectance spectroscopy, the same Xe lamp was used with the light beam directed at an angle of 65° with reference to the surface normal. During the evaporation process, the spectral intensity *S*(*h*ν, *t*) measured after reflection at the sample surface increases or decreases, depending on the optical properties of the evaporated molecules and the film thickness. Assuming a constant intensity of the incoming light, these signals can be related to changes in the reflectivity *R*(*h*ν, *t*) ∝ *S*(*h*ν, *t*). After the reflection of the light at the sample surface, the beam is split into a p-polarized and an s-polarized part by a Glan–Thompson prism and is focused by a lens into two separate spectrometers (STS-UV from Ocean Optics). The spectrometers span a spectral range from 190 to 650 nm (corresponding to photon energies between 6.52 and 1.91 eV), but due to the transmission of the viewports and other optical components, there is almost no detectable intensity above *h*ν = 4 eV. The spectral resolution is 1.5 nm. In Figure 4c,d, we show only the incremental changes in the optical data following the definition^20^ of the so-called DDRS signal
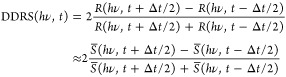
2Here, Δ*t* is the time interval needed for the deposition of approximately 1/20 monolayer. The overline above the spectral intensity *S* indicates an averaging of all spectra in the intervals [*t* – Δ*t*/2; *t*[ and ]*t*; *t* + Δ*t*/2] to improve the signal-to-noise ratio.

## 3 Results and Discussion

First, we want to discuss the data acquired with PEEM and DDRS during the deposition of a 2.66 ML thick film on a clean Au(111) surface. For each PEEM image taken, a histogram of the electron yield EY(*t*, *x*, *y*) (see eq 1) was calculated, which is shown as background in Figure 4a in a false color representation as a horizontal line. The red dots mark the mean values of the respective PEEM image. In Figure 4b, the standard deviation related to the image intensity (σ_pixel_) and the one related to the morphology of the image (σ_image_) are shown following a procedure based on image averaging as outlined in Part I. Figure 4c,d shows the data for incremental changes in the optical reflectance for p- and s-polarized light.

Upon opening the shutter, a sizable amount of pixels in the normalized images become immediately darker compared to their initial value, corresponding to the bare surface (see the distribution of pixel intensity in Figure 4a). The reason for this is that defects (or dust particles) on the surface are decorated first and, therefore, quickly appear as dark regions in the images. In agreement to this, the standard deviation related to the image homogeneity increases.

Within the next 250 s (corresponding to about a quarter of the later defined equivalent of a monolayer), the image intensity does not change significantly (corroborated by an almost constant standard deviation ), whereas the reflectivity for p- and s-polarized light with energy *h*ν ≥ 2.5 eV decreases significantly for both. Such a behavior was already observed for the case of (NapNC)AuCl on Au(110) surfaces (see the Results and Discussion section in Part I) and can be attributed to the presence of a 2D molecular gas consisting of flat-lying (NapNC)AuCl monomers.

This is also confirmed by the STM and LEED data shown in Figure 2b. Here, we stopped the deposition at a nominal (NapNC)AuCl coverage of Θ = 0.25 ML and subsequently annealed the sample for 5 min at *T* = 363 K. Further down, we define the nominal monolayer coverage as half of the molecular material of the two-molecular-layer-thick wetting layer. The STM image reveals that the herringbone reconstruction is still present and in principle unchanged. Nevertheless, we were not able to image it with atomic resolution as the STM tip changed frequently due to the presence of the adsorbed and highly mobile molecules in a 2D gas phase. The hcp domains are hardly distinguishable from the domain walls, which may indicate a longer dwell time of the molecules in these areas. Nevertheless, the corrugation of this STM image compared to the one of the bare surface (see Figure 2a) is not affected.

Whereas the reconstruction of the bare Au(111) surfaces causes characteristic satellite spots in the LEED pattern around the fundamental spots of the truncated bulk structure, these satellite spots vanish almost completely upon deposition of about 0.25 ML of (NapNC)AuCl. By tilting the sample intently, we were able to image the vicinity of the (0,0) spot: a faint halo around this spot indicates a molecule–molecule distance of roughly 1.2 nm. This value corresponds to the longest dimension of a (NapNC)AuCl monomer. The halo can therefore be interpreted as the structure factor of individual molecules in a weakly ordered (2D gas) phase. Due to the low kinetic energy of the electrons used (*E*_kin_ = 40.6 eV), the LEED pattern becomes extremely surface sensitive (see universal curves published by Seah and Dench).^24^ In conclusion, STM and LEED data suggest a 2D molecular gas phase at a coverage of Θ = 0.25 ML.^25,26^

As already reported in Part I for (NapNC)AuCl on Au(110), a density of the 2D molecular gas of around 0.25 ML seems to be critical for the formation of dimers on the surface. Indeed, for coverages above 0.25 ML, the optical reflectivity of the p-polarized light increases, whereas the s-polarized light stays roughly constant. This indicates that the molecular transition dipole is now pointing out of the surface plane, induced by the formation of dimers in a crossed swords configuration. Up to Θ ≈ 0.9 ML, there is a steady increase of the mean electron yield without visible structure formation (see Figure 3c). MEY and σ_pixel_ then reach a maximum followed by a kind of plateau up to Θ ≈ 1.2 ML. Similarly, σ_image_ exhibits a kink at Θ ≈ 0.9 ML and continues to increase with a smaller slope until Θ ≈ 1.2 ML. This increase comes from the inhomogeneity of the image, which is clearly visible from the PEEM image at 1.16 ML (Figure 3d) as bright and dark stripes. The fact that the elongated structures form only along one of the three equivalent crystalline ⟨*x*, *y*, and *z*⟩ directions of the Au(111) surface might be related to a miscut of the surface plane. In fact, the structure may be guided by the preferential orientation of the step edges orthogonal to the miscut direction.

At the same time, i.e., between Θ = 0.9 and 1.2 ML, the maximum of the reflectivity shifts from 3.3 to 3.45 eV. Then the signal amplitude drops to zero. Only after deposition of more than an equivalent of 2 ML do well-defined (in shape and contrast) elongated dark structures appear in the PEEM images (Figure 3f–h) and, at the same time, the p-polarized DDRS signal increases again. The continuous increase of the lateral size of these structures from Figure 3f–h is more typical for a 2D island growth rather than a sudden onset of a 3D crystallite growth. Therefore, we assume that in Figure 3f, the second layer is just closed, and the third layer starts to be filled with molecules. In accordance with this, σ_image_ exhibits a local minimum at the same coverage. Taking all this evidence and also the STM data into account, we attribute the coverage reached after a deposition time *t* = 2030 s to be an equivalent of 2 ML.

Note that the PEEM transients have another maximum at Θ ≈ 1.4 ML. A corresponding PEEM image is shown in Figure 3e. It is likely that this marks condensation of the molecules in the second layer from a 2D molecular gas phase covering the first monolayer of (NapNC)AuCl on the surface.

**Figure 5:**
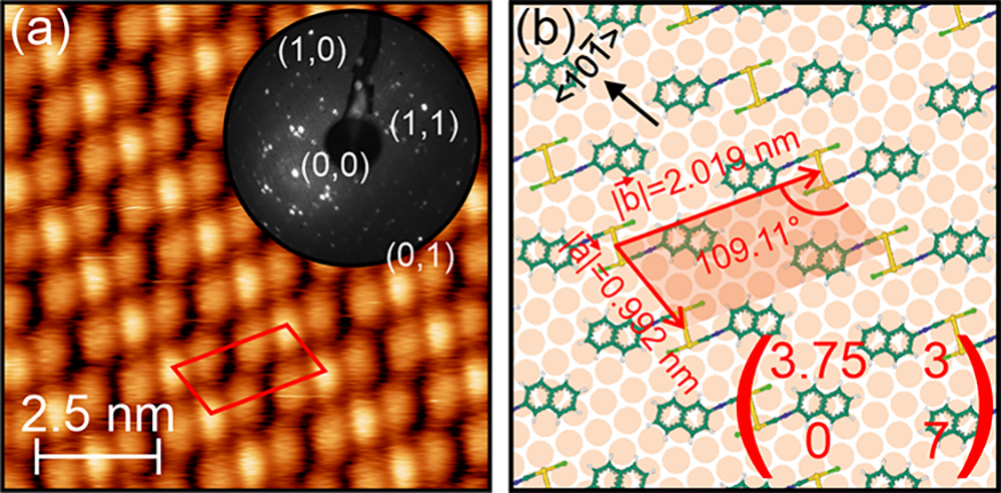
(a) STM image of a Au(111) surface after the deposition of about 0.9 ML (NapNC)AuCl and subsequent annealing at 363 K for 5 min. The image size is 10 × 10 nm^2^. The image was acquired with *U*_sample_ = 1 V and *I*_T_ = 1 nA. The LEED pattern shown as an inset was recorded with an electron energy of 40 eV in an off-axis geometry. (b) Corresponding model (*M*_1_ given in eq 3) of the surface unit cell.

**Figure 6:**
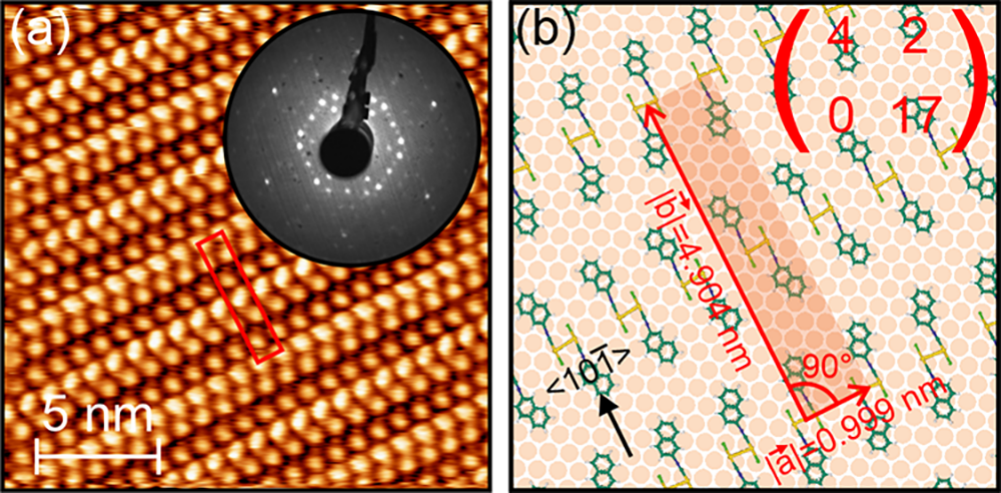
(a) STM image of a Au(111) surface after the deposition of about 1.1 ML of (NapNC)AuCl and subsequent annealing at 363 K for 5 min. The image size is 20 × 20 nm^2^. The image was acquired with *U*_sample_ = 100 mV and *I*_T_ = 1 nA. The LEED pattern shown as the inset was recorded with an electron energy of 22.6 eV in an off-axis geometry. (b) Corresponding model (*M*_2_ given in eq 4) of the surface unit cell.

To understand the complex transients obtained with PEEM and pol-DDRS, we stopped deposition at selected characteristic points and performed additional STM and LEED experiments. For coverages slightly below and above 1 ML, we found two, well-ordered characteristic structures, which seem to coexist on the surface and differ in shape and packing density (see Figures 5 and 6).

Although both structures were present after deposition, the denser structure *M*_1_ was found to dominate the surface after annealing (see also the Supporting Information). This indicates that phase *M*_1_ is the thermodynamically more stable variant. Therefore, we want to describe it first. STM images of the structure *M*_1_ like the one in Figure 5a show characteristic groups of three protrusions oriented parallel to a close-packed ⟨101̅⟩ direction of the substrate and stacked along an oblique angle of ±109° to this direction. The central protrusion appears to be highest. The outer ones are symmetrical, i.e., they have the same distance to the central protrusion and the same apparent height. We suggest that such a chain corresponds to a “crossed swords” dimer (see Figure 1) known from the arrangement of the molecules in the crystalline bulk.^10^ Neglecting all electronic interaction between the molecules and the surface and the resulting distortions of the dimer structure, the dimer extents from the atoms closest to the surface to the ones farthest away, 0.508 nm (see Figure 1b). The data obtained from the STM experiments suggest that the molecules are still intact after evaporation. Thus, the unit cell marked by the red lines in Figure 5 contains two molecules. A possible arrangement of the molecules on the surface can be seen in the model depicted in Figure 5b. Given the three equivalent directions of ⟨101̅⟩ within a (111) surface and the fact that one axis of the molecular unit cell is aligned with them, one would expect six different growth domains. These six domains were confirmed with LEED (see the inset of Figure 5a and the Supporting Information). The detailed analysis of the LEED data suggests the following epitaxial matrix
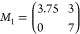
3Although the matrix implies that the structure is only simple commensurate across the dimer rows, we were unable to observe any height modulation along the stacking direction of the dimers. The structural model *M*_1_ in Figure 5b proposes that the center of mass of the cross swords dimers adsorbs on bridge sites with different “orientations”. Orienting the aurophilic bond along the ⟨12̅1⟩ directions of the surface would allow for the required local 2-fold symmetry so that the naphthyl groups would also experience an identical local environment. Since such an arrangement, although not simple commensurate, allows for “similar” environments of the dimers on the surface, there was no detectable (apparent) height modulation along the dimer rows.

In addition, we did not observe any height modulation due to a herringbone reconstruction of the surface preserved beneath the first layer of condensed (NapNC)AuCl dimers. While the molecular corrugation (apparent height) in Figure 5 (as well as in Figure 6 for structure *M*_2_) is about 0.1 nm, the height difference between soliton walls and hcp or fcc regions of the herringbone reconstruction is only 0.014 nm. Yet, such a height modulation should be detectable in STM. Since this was not the case, we infer that the herringbone reconstruction was lifted either already during dimer formation or during condensation of these dimers into the structures *M*_1_ or *M*_2_.

Taking into account its internal degrees of freedom, the (NapNC)AuCl monomer is achiral in the gas phase. Adsorption on a surface makes the monomer chiral: there are two mirror configurations that cannot be mapped to each other by a simple azimuthal rotation, i.e., around an axis orthogonal to the surface plane. The interaction with the surface prevents the other two possible rotations of the rigid molecule. Two monomers with the same handedness can form a dimer that is also chiral. Due to the 3D bending of the molecules in the dimers on the surface, there is some similarity to a clockwise and anticlockwise propeller considering a rotation axis centered at the aurophilic bond. The arrangement of the dimers within the unit cell, given by the matrix *M*_1_, allows only one handedness within each mirror domain.

In contrast to structure *M*_1_ (see eq 5), the STM images of the second structure *M*_2_ clearly reveal a higher-order periodicity between adjacent rows of dimers formed along the ⟨110⟩ direction of the substrate. As can be seen in Figure 6a, the dimers within one row are rotated with respect to the stacking direction to one side. In the adjacent row, they are rotated to the other side.

Assuming a similar adsorption geometry as for structure *M*_1_, this behavior can be explained by the handedness of the “crossed swords” dimers on the surface. Whereas one row of dimers is formed by right-handed dimers, the adjacent row (in the ⟨101̅⟩ direction) will be left-handed. As a consequence, the unit cell formed by (NapNC)AuCl does not contain a single dimer but two dimers with opposite handedness. Due to the steric repulsion of the outward pointing naphthyl groups, this arrangement of dimers is more space-consuming, i.e., the density of this (dilute) phase is expected to be smaller. The analysis of the STM and LEED data suggest the following epitaxial matrix
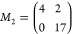
4corresponding to a rectangular unit cell containing two dimers (of opposite handedness) or four molecules. Note that the occupied surface area per dimer (footprint) in the two structures *M*_1_ and *M*_2_ are det *M*_1_ = 26.25 and det *M*_2_/2 = 34 times the area of the substrate unit cell confirming the lower packing density of phase *M*_2_ compared to phase *M*_1_. A summary of the characteristics of these structures found on Au(111) and a comparison of those found on Au(110) (Part I of the study—see ref (8)) is given in Table 1. Nevertheless, the structure *M*_1_ does not reach the density of the corresponding projective bulk structure (bulk III in Table 1) nor that given by the smallest rectangle containing a dimer (lying flat on the surface) as shown in Figure 1a. In comparison, the structure *M*_2_ contains only half of the number of molecules per unit area. Therefore, *M*_2_ is only a transient structure that does not correspond to an absolute energy minimum but only to a local one.

**Table 1:** Properties of the Two Structures of (NapNC)AuCl Found on Au(111) and Au(110)^8^^,^^a^

surface	Au(111)		Au(110)		
structure					(9 × 1)
orientation	flat-lying				upright-standing
|*a⃗*|	0.992 nm	0.999 nm			0.408 nm
|*b⃗*|	2.019 nm	4.904 nm	1.891 nm	1.999 nm	2.592 nm
∠(*a⃗*, *b⃗*)	109.11°	90°	95.05°	114.09°	90°
*A*	1.890 nm^2^	4.897 nm^2^	1.882 nm^2^		1.058 nm^2^
*Z*	2	4	2		4
*A*/*Z*	0.945 nm^2^	1.224 nm^2^	0.941 nm^2^		0.264 nm^2^
ϱ	1.058 nm^–2^	0.817 nm^–2^	1.063 nm^–2^		3.781 nm^–2^

a *a⃗* and *b⃗* denote the molecular unit cell vectors. *A* is the area of the superstructure unit cell, and *Z* is the number of molecules in the superstructure unit cell. ϱ is the density of molecules in a layer on the surface per unit area (nm^2^).

### 3.1 Semiquantitative Growth Model

**Figure 7:**
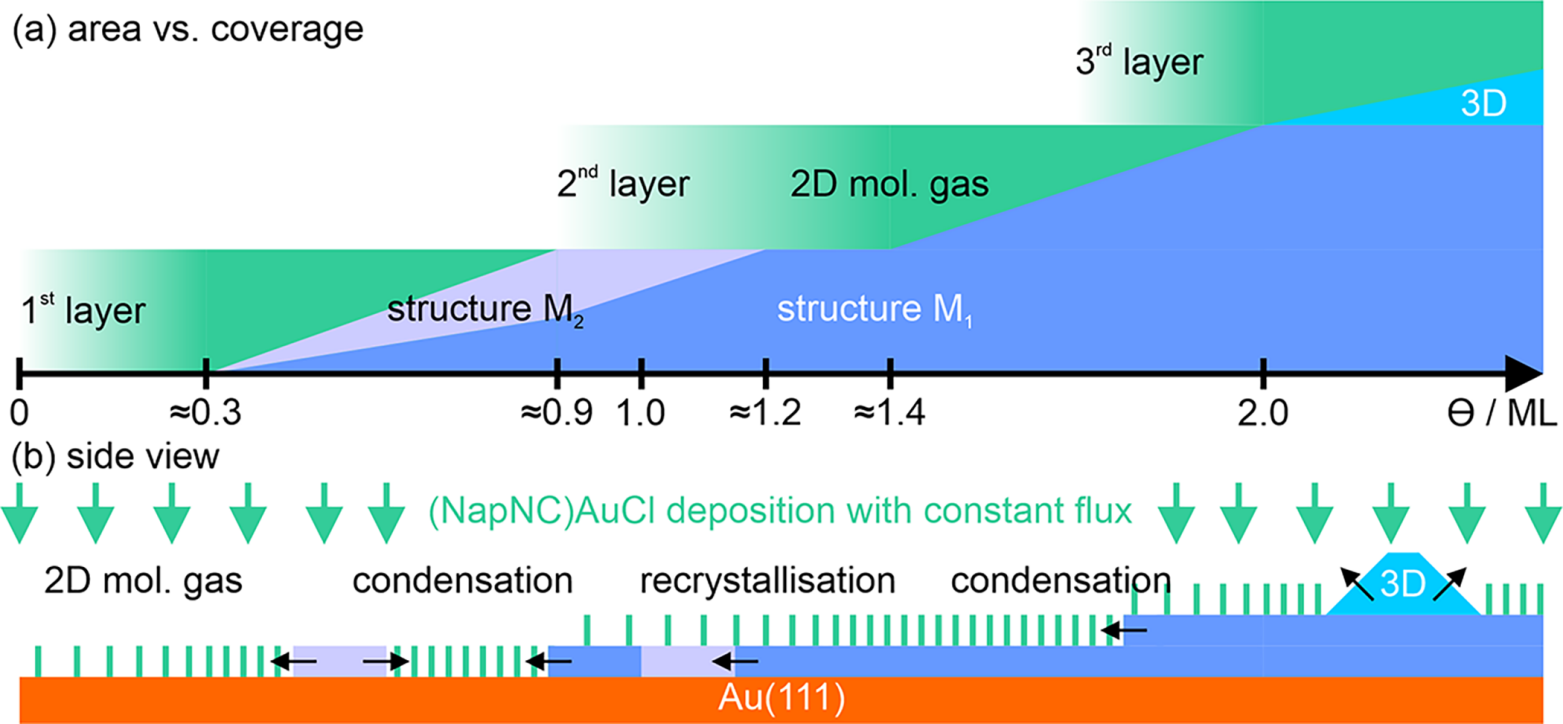
(a) Schematic evolution of the surface area covered by different phases (2D gas phase, structures *M*_1_ and *M*_2_, and 3D crystallites) in the different layers on the Au(111) substrate as a function of the total (NapNC)AuCl coverage. (b) Symbolic representation in a “side view”.

To explain in detail the PEEM transient with its three maxima and evolution of the DDRS data, we developed a semiquantitative model, which is depicted in Figure 7. The first maximum of the PEEM transient and a kink of the standard deviation σ_image_ is found for Θ ≈ 0.9 ML, so before a coverage of one monolayer is reached. Remember that the definition of the monolayer equivalent was defined as half the time needed to complete the 2 ML thick wetting layer. We can assume that the wetting layer is equivalent to two monolayers of the denser structure, *M*_1_ (shown in Figure 5). Therefore, we can expect that the surface is already covered completely with a mixture of the two phases, *M*_1_ and *M*_2_, at a coverage of Θ ≈ 0.9 ML.

Both phases consist of (NapNC)AuCl dimers, which are most likely already formed at a stage where only a 2D molecular gas phase is present on the surface. Assuming that these “crossed swords” dimers condense with the same probability into either structure *M*_1_ or *M*_2_, the relative area covered by the two structures is just given by their relative 2D molecular densities ϱ_*M*_2__ and ϱ_*M*_1__. Therefore, it follows
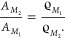
5The sample surface with area *A* is completely filled with molecules if
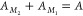
6Therefore, the relative surface coverage with the two structures can be estimated from the densities given in Table 1
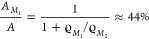
7
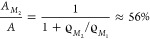
8Finally, we want to estimate the coverage Θ (in multiples of a complete monolayer given by the denser structure *M*_1_), when the surface is completely covered with only the condensed structures *M*_1_ and *M*_2_ and there is no 2D molecular gas left in the first layer:
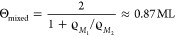
9This nicely corresponds with the first maximum of the mean electron yield at Θ = 0.9 ML. As the surface is already covered with a monolayer of molecules, the further changes in the work function will be less dramatic. Consequently, there is now a plateau of the MEY until the areas covered by structure *M*_2_ are fully converted to the denser *M*_1_ phase. This transformation is likely triggered by molecules in the second layer being less strongly bound to the substrate, thus favoring incorporation into the first layer by compressing its structure. The reorganization of the first monolayer is also evident in the DDRS data. The maximum of the changes of the optical reflectivity changes from 3.30 to 3.45 eV. Indeed, the structural changes in the first molecular layer will affect the electronic structure of the substrate and, hence, the energy of the observed substrate related optical transitions.

The minimum required material to be deposited to transform the dilute phase *M*_2_ into the dense *M*_1_ structure would correspond to 0.13 ML, but the plateau of the PEEM transient in Figure 4a,b spans almost 0.3 ML. Most likely, only molecules impinging the uncompressed areas (covered with phase *M*_2_) will be incorporated into the first layer and contribute to the transformation from phase *M*_2_ into denser phase *M*_1_. Molecules adsorbed on areas already covered with structure *M*_1_ will form a 2D molecular gas phase in the second layer. The molecules in the second layer no longer change the work function but can reduce the electron yield of the photoelectron emitted from the substrate by scattering.^27^ The enhanced contrast or increased σ_image_ between Θ = 0.9 and 1.2 ML supports this scenario.

The drop of the MEY and σ_image_ at Θ ≈ 1.2 ML indicates that the transformation from phase *M*_2_ into the denser phase *M*_1_ in the first layer is complete. As a result, the surface is imaged by PEEM with no structures and the standard deviation σ_image_ decreases.

Structures become visible again in the PEEM images at Θ ≈ 1.4 ML (see Figure 3e). Now, the molecular 2D gas in the second layer starts to condense into densely packed 2D islands. These islands in the second layer exhibit a stronger scattering for the photoelectrons excited from the substrate, which now have to pass through two densely packed layers.^27^ Therefore, the 2D islands appear darker in the PEEM images. On the other hand, the sudden condensation could reduce the (supersaturated) density of the molecular 2D gas just before condensation so that less scattering of photoelectrons occurs in regions still covered by the 2D gas phase. This effect could therefore be responsible for the intermittent maximum of the mean electron yield at ≈1.4 ML.^28,29^ As the second layer becomes more and more covered by a condensed (dense) structure, the mean electron yield and the standard deviation related to the image homogeneity decrease again.

### 3.2 Comparison of (NapNC)AuCl on Au(110) and Au(111)

Table 1 summarizes and compares our finding for the deposition of (NapNC)AuCl on the isotropic Au(111) and anisotropic Au(110) surfaces. Before (NapNC)AuCl deposition, we confirmed for both surface orientations that they were reconstructed, i.e., the Au(111) surface showed a herringbone reconstruction and the Au(110) a (1 × 2) missing row structure. Upon (NapNC)AuCl adsorption, the reconstructions are lifted in both cases. This implies not only a certain mass transport of the expelled Au atoms but also indicates a strong interaction between the molecules of the first layer and the surface atoms.

On the Au(111) surface, (NapNC)AuCl is exclusively found in a flat-lying dimer (“crossed swords”) configuration, whereas we could observe also an (almost) upright-standing configuration of (NapNC)AuCl monomers on Au(110) surfaces for nominal coverages close to 1 ML. For the flat-lying “crossed swords”, we suggest that the main contribution to the binding energy originates from the overlap of the electronic π system of the naphthyl groups with the electronic states of the surface atoms. Still the lateral order is dominated by the aurophilic interaction between the molecules forming the characteristic “crossed swords” dimers.

On both surfaces, we found similar, but not identical unit cells for a coverage of about one monolayer: the  structure on the Au(111) surface corresponds to the  structure found on the Au(110) surfaces. As discussed in Part I, this is an “in phase” alignment of rows along the ⟨11̅1⟩ direction of the substrate, i.e., -XX- or -YY- sequence of rows. Due to the weak interaction between the rows, this phase on the Au(110) was considered to be stabilized by aurophilic interactions and energetically similar to the “out-of-phase” configuration, in which alternating X and Y rows were the packing motif.

Finally, it is also interesting to note that the unit cell vector *a⃗* of the  and  on Au(110) surfaces and the respective vector of the  on Au(111) ones have exact the same length, namely, |*a⃗*|=*a*_Au_ with *a*_Au_ = 0.408 nm.

## 4 Summary and Conclusions

We have studied the growth of (NapNC)AuCl on the isotropic Au(111) surface and compare the results to those obtained on the anisotropic Au(110) surface. The “crossed swords” structure characteristic of (NapNC)AuCl was found on both surfaces, which confirms the presence of an aurophilic interaction not only in the bulk but also in a thin film phase of (NapNC)AuCl. In the case presented here of Au(111) substrates, the detailed analysis of the growth process reveals a complex interplay between a 2D molecular gas phase and two condensed structures with different density. The experiments suggest that the two condensed phases are energetically almost equally stable if the number of excess molecules in a second layer is small. Only when the first layer is completely filled with molecules in a condensed structure does the compression of this layer give rise to an energy gain. This indicates that the molecule–molecule interaction is less dominant than the molecule–substrate interaction. Therefore, after completion of the two-layer-thick wetting layer, the growth continued in a 3D fashion.

Our data for the isotropic Au(111) surface confirm only flat-lying “crossed swords” dimers on the surface for all coverages. An upright adsorption geometry was found only for the less densely packed Au(110) surface at high (NapNC)AuCl coverage. The lower packing density of the substrate atoms in the Au(110) surface along the ⟨101̅⟩ direction results in a higher electronic surface corrugation turning the molecules finally upward. Whether this is an aurophilic interaction cannot be answered with the presented data. Definitely, the interaction between the (NapNC)AuCl dimers in the first layer and the uppermost layer of the gold substrates is strong enough so that the respective reconstructions of the bare surfaces are lifted.

## Supporting Information Available

Differential reflectance spectroscopy, temperature- and voltage-dependent STM, and position-dependent LEED images (PDF)
