# Supplementary material for: Aurophilic Molecules on Surfaces. Part II. (NapNC)AuCl on Au(111)
Source: ACS Omega. 2023 Oct 6;8(41):38083–91. doi: 10.1021/acsomega.3c04152 (PMC10586446; doi:10.1021/acsomega.3c04152)
Supplement: Supplementary file 1 — ao3c04152_si_001.pdf [file ao3c04152_si_001.pdf]

Aurophilic Molecules on Surfaces - Part II: (NapNC)AuCl on Au(111) –supporting information–

Thorsten Wagner,*,† Michael Györök,† Sebastian Wolfmayr,† Petra Gründlinger,† Uwe Monkowius,‡ and Peter Zeppenfeld†

†*Johannes Kepler University, Institute of Experimental Physics, Surface Science Division, Altenberger Straße 69, 4040 Linz, Austria*

‡*Johannes Kepler University, School of Education, Chemistry, Altenberger Straße 69, 4040 Linz, Austria*

E-mail: thorsten.wagner@jku.at

1 Differential Reflectance Spectroscopy

Figure 4 of the main article shows the incremental changes of the reflectivity between 2 eV and 4 eV. Similar results were obtained for the deposition of (NapNC)AuCl on Au(110) surfaces (see ref 1). Here we want to rationalize, at least qualitatively, the amplitude and sign of the observed spectral changes.

To this end, we have modeled the optical reflectance for a so-called three-layer-system consisting of (1) vacuum with refractive index $N_1 = n_1 = 1$, (2) an organic thin film with anisotropic refractive index $N_2 = (N_{2x}, N_{2y}, N_{2z})$ and thickness d, and (3) a thick gold substrate with complex refractive index $N_3 = n_3 + \imath k_3$ as tabulated in ref 2. A sketch of the model is shown in the insets of Figures S1 and S2.

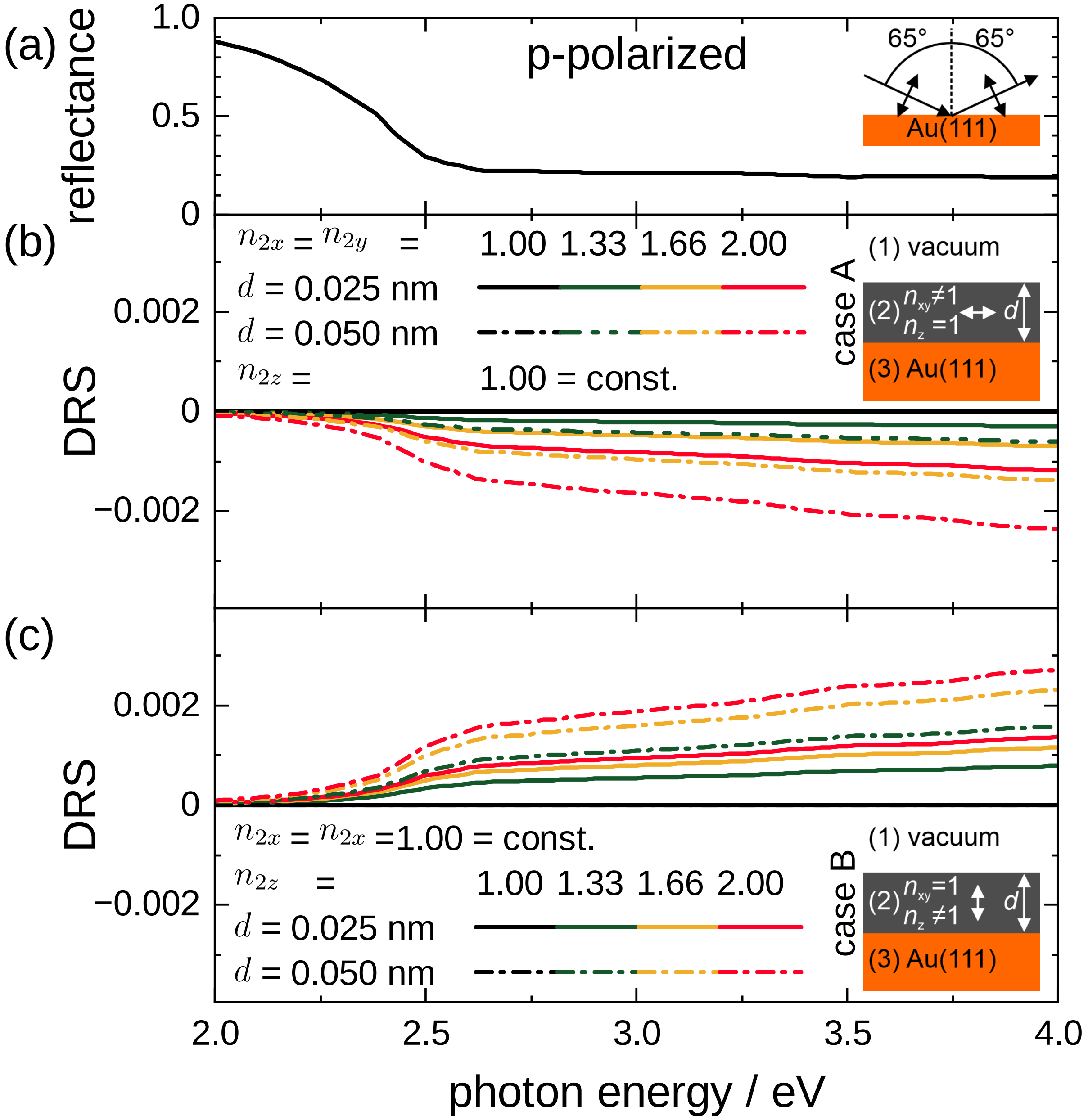

Figure S1: (a) simulated reflectance of the bare Au(111) surface for p-polarized light incident on the surface at an angle of 65° to the surface normal. Simulated DRS signals for p-polarized light caused by an additional anisotropic layer (2) with varying in-plane (b) and out-of-plane (c) components of the refractive index n_2. The simulation was carried out for two different thicknesses d of the intermediate layer.

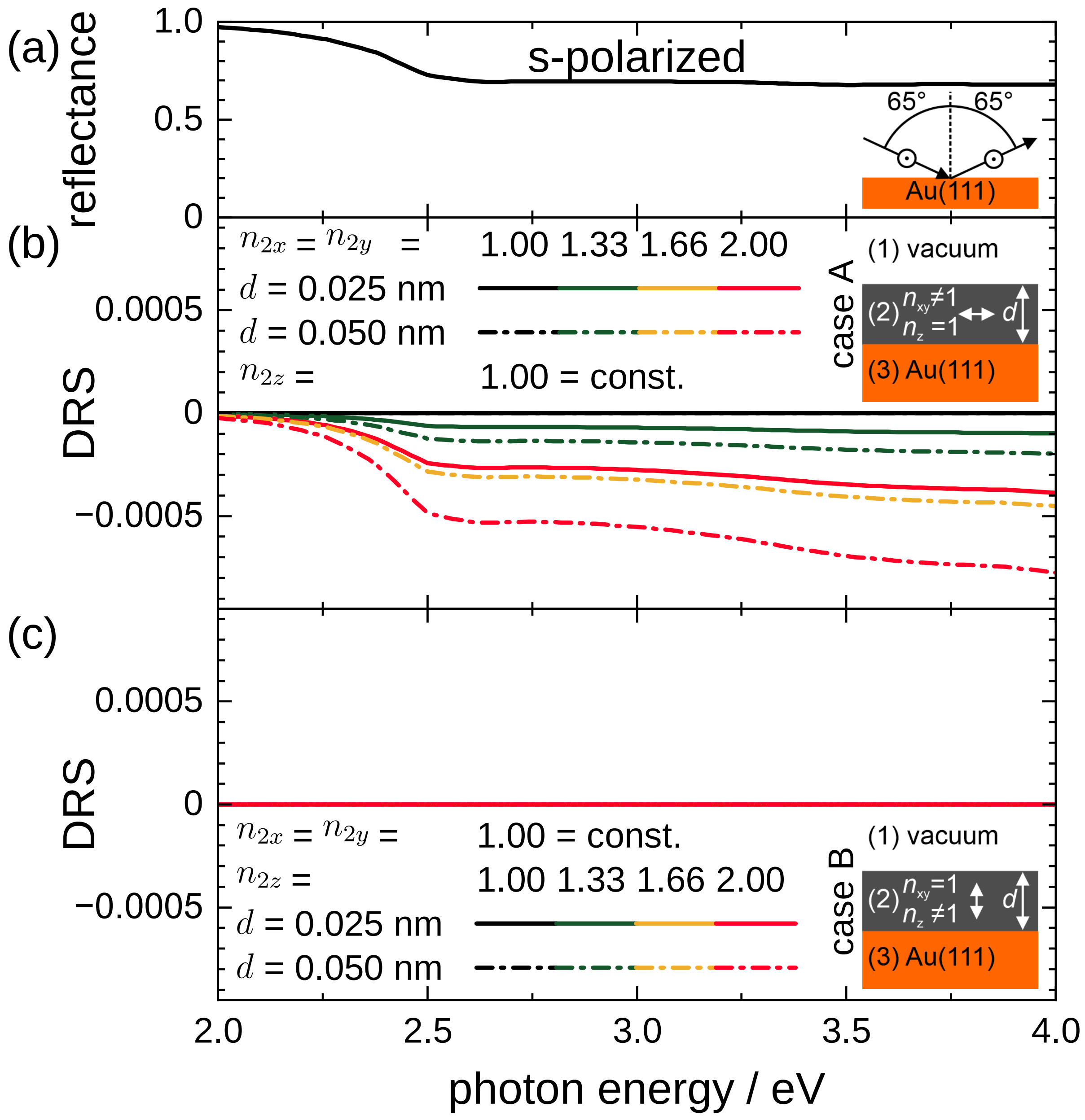

Figure S2: (a) simulated reflectance of the bare Au(111) surface for s-polarized light incident on the surface at an angle of 65° to the surface normal. Simulated DRS signals for s-polarized light caused by an additional anisotropic layer (2) with a varying in-plane (b) and out-of-plane (c) components of refractive index n_2. The simulation was carried out for two different thicknesses d of the intermediate layer.

As reported by Hobbollahi and coworkers in ref 3, the optical transitions of (NapNC)AuCl are located in the UV range ($E \geq 4\,\mathrm{eV}$, $\lambda \leq 300\,\mathrm{nm}$). Away from the absorption resonances, the refractive index is essentially real valued ($N_{2i} = n_{2i} + \imath k_2 \approx n_{2i}$ with $i \in \{x, y, z\}$) and its wavelength dependence can be described by a Cauchy term:

$$n_{2i}(\lambda) = B_{0i} + \frac{B_{1i}}{\lambda^2} \tag{S1}$$

or (in a first approximation over a limited spectral range) simply by a constant value $n_{2i} \geq 1$.

In fact, ref 4 reports optical data for common organic molecules containing naphthyl groups. The complex refractive index for these molecules are almost featureless in the visible range, besides a steady, but small increase of n and k towards the UV. Moreover, n is about a factor 30 larger than k. Therefore, our subsequent assumption of constant, real-valued components for the (anisotropic) refractive index N_2 with values n_{2i} in the interval between 1 and 2 seems well justified.

According to Azzam (see ref 5 and there in particular chapter 4.7.3.4), for the case of a biaxially anisotropic film (2) on an isotropic substrate (3) in an isotropic ambient medium (1), where the principal axes x and y of the biaxial film lie in the film plane, while the third axis (z) is perpendicular to the film plane (i.e., parallel to the surface normal) the reflection coefficients for s- or p-polarized light for the entire three-layer-system are as follows:

$$r_{123p} = \frac{r_{12p} + r_{23p}e^{\imath 2\beta_p}}{1 + r_{12p}r_{23p}e^{\imath 2\beta_p}} \tag{S2}$$

$$r_{123s} = \frac{r_{12s} + r_{23s}e^{\imath 2\beta_s}}{1 + r_{12s}r_{23s}e^{\imath 2\beta_s}} \tag{S3}$$

If the scattering plane is located in the (x, z)-plane, then the so-called phase thicknesses β_s

and β_p are defined as follows:

$$\beta_p = 2\pi \frac{d}{\lambda} \frac{N_{2x}}{N_{2z}} \left(N_{2z}^2 - N_1^2 \sin^2\theta_1\right)^{1/2} \tag{S4}$$

$$\beta_s = 2\pi \frac{d}{\lambda} \left(N_{2y}^2 - N_1^2 \sin^2\theta_1\right)^{1/2} \tag{S5}$$

The Fresnel equations give the reflection coefficients r_{12s} and r_{12p} at the interface between layers 1 and 2

$$r_{12p} = \frac{N_{2x}N_{2z}\cos\theta_1 - N_1\left(N_{2z}^2 - N_1^2\sin^2\theta_1\right)^{1/2}}{N_{2x}N_{2z}\cos\theta_1 + N_1\left(N_{2z}^2 - N_1^2\sin^2\theta_1\right)^{1/2}} \tag{S6}$$

$$r_{12s} = \frac{N_1\cos\theta_1 - \left(N_{2y}^2 - N_1^2\sin^2\theta_1\right)^{1/2}}{N_1\cos\theta_1 + \left(N_{2y}^2 - N_1^2\sin^2\theta_1\right)^{1/2}} \tag{S7}$$

In the above equations, θ_1 denotes the angle between the light beam in the ambient medium (1) and the direction normal to its interface with layer 2. The equations above can also be used to calculate the reflection coefficients $r_{23s} = -r_{32s}$ and $r_{23p} = -r_{32p}$ between layers 2 and 3 by replacing the index 1 by 3 and using Snell's law $N_3 \sin\theta_3 = N_1 \sin\theta_1$. Likewise, the reflection coefficients from the bare surface r_{13s}, and r_{13p} can be obtained using the same formula. In the latter case, the Fresnel equations simplify to the well known form for the interface between two optically isotropic media.

Note that on the Au(111) substrate with an in-plane three-fold rotational symmetry, the (NapNC)AuCl overlayer will have a net laterally isotropic refractive index, i.e., $N_{2x} = N_{2y}$, whereas on the Au(110) substrate the molecules in the overlayer are uniaxially aligned[1] and N_{2x} will generally differ from N_{2y}.

In a next step, the reflectances for p- and s-polarized light are obtained from the corresponding reflection coefficients via $R_s = |r_s|^2$ and $R_p = |r_p|^2$, respectively. The reflectances of the bare Au(111) surface $R_{13p} = |r_{13p}|^2$ and $R_{13s} = |r_{13s}|^2$ are shown in the panels (a) of Figures S1 and S2, respectively. For N_3, we used a linear interpolation of the data-set of Johnson and Christy for polycrystalline gold published in the refractive.index database.[6] Since

the angle of incidence, $\theta_1 = 65°$, is close to the Brewster angle for gold, the reflectance for s-polarized light is significantly higher than for p-polarized light, especially above $\sim 2.5\,\mathrm{eV}$.

To explore the effect of the (NapNC)AuCl overlayer, we calculated the DRS spectra according to $DRS(d) = (R_{123} - R_{13})/R_{13}$ for both p- and s-polarization and a given layer thickness d. Since the thickness of the (NapNC)AuCl overlayer in all our experiments is of the order of 1 nm or below, the DRS spectra are strictly linear in d since interference effects between the light reflected at the interfaces between layers 1 and 2 and between layers 2 and 3, respectively, are negligible for $d \ll \lambda$. Conequently, we can directly compare the calculated DRS spectra, $DRS(d)$, to the measured DDRS spectra at time t and increment Δt, if we choose d such that it corresponds to the thickness of the overlayer deposited in the time interval Δt and values for the refractive index N_2 characteristic of the molecules added in the growth stage at deposition time t. Reversely, the calculated spectra $DRS(d)$ are identical to the DDRS spectra obtained for the incremental growth of an overlayer (with fractional thickness d and momentary refractive index N_2), as long as the incremental thickness d as well as the *total* thickness of the overlayer are both much smaller than the wavelength λ of the probing light.

From the adsorption geometry and the height difference between the lowest hydrogen atoms and the chlorine atoms in the (NapNC)AuCl molecule marked in Figure 1 of the main article, we infer an increase of the overlayer thickness of about $d = 0.025\,\mathrm{nm}$ over the time step between consecutive DDRS spectra Δt, which corresponds to an increment of the coverage $\Delta\Theta \approx 0.05\,\mathrm{ML}$ for the relevant adsorption geometry. To estimate the influence of the thickness increment d, we also ran our simulation for $d = 0.05\,\mathrm{nm}$. As can be seen from Figures S1 and S2, the changes in the differential reflectivity for both p- and s-polarized light scale linearly with the thickness d (as expected for $d \ll \lambda$).

For our simulation, we considered two basic cases: (A) the thin layer has only an in-plane isotropic contribution, i.e., the z component of the refractive index, n_{2z} is set to 1, versus (B) the thin layer has only an out-of-plance contribution, i.e., the in-plane components

of the refractive index, n_{2x} and n_{2y} are both set to 1. In both scenarios, we varied the complementary component of the refractive index ($n_{2x} = n_{2x}$ in case A and n_{2z} in case B) over a range between 1 (no effect) and 2. The data clearly show a nonlinear dependence on the respective component of n_2.

Case B is certainly the simpler one: The s-polarized light is not affected by an out-of-plane component of the refractive index since only the p-polarized light probes the n_{2z} component. The corresponding results of the simulation for case B are shown in Figures S1c and S2c. As expected, the DRS signal for s-polarized light is a flat line equal to 0. The p-polarized light corresponds to an increased reflectivity of the sample. Although the assumed (real) refractive index of the organic layer shows no dispersion, i.e., is constant across the entire photon energy range shown, the DRS signal changes most, where the reflectance of the bare sample is small. Therefore, the resulting DRS signal resembles the shape of the reflectance of the bare gold surface (see Figures S1a and S2a). A refractive index with mainly an out-of-plane component should be characteristic for (NapNC)AuCl dimers on the surface. In such a configuration, the molecules are not only tilted with respect to the surface but also slightly bent. The resulting dipole layer might thus also affect the charge distribution of the topmost layer of the substrate.

Case A represents (NapNC)AuCl molecules with only an in-plane component of the refractive index. In addition, the refractive index is assumed to be isotropic in the (x, y)-plane. As mentioned earlier, this is certainly valid for molecules adsorbed on the optically isotropic Au(111) surface. The calculated DRS spectra reveal a decrease of the reflectance for both p- and s-polarized light, as shown in Figures S1b and S2b. Note that the amplitude of the change in reflectivity for p-polarized light is about a factor of four larger than that for s-polarized light. This results from the angle of incidence being close to the Brewster angle. Such a difference in the signal amplitudes was also observed in the experiment (see Figures 4c and 4d of the main article). As in case B, the spectral lineshape follows that of the reflectance of the bare surface. However, the reflectance is now decreasing and not

increasing as in case B. Since we can reproduce the correct order of magnitude of the changes as well as their sign, the assumed combinations of layer thickness and refractive index of the organic layer seem to describe the real system rather well.

2 Temperature and Voltage Dependent STM

Figure S3 shows STM images taken after deposition of $\Theta \approx 1.2\,\mathrm{ML}$ (NapNC)AuCl on a Au(111) single crystal held at room temperature. In both cases, the deposition was stopped immediately after the plateau of the PEEM transient (see Figure 4a of the main article). The surface shown in Figure S3a was imaged without further heat treatment at room temperature: two domains are visible. The stacking direction of the dimers along the short unit cell axis differs bei about 22° between the two domains. This indicates that the imaged surface is covered by the M_1 structure as discussed in the main article. It also shows that the domains extend over more than 100 nm in each of the two dimensions on the surface. The noise in the image indicates that molecules are present in the second layer forming a 2D gas phase.

Such movement of molecules in a 2D molecular gas phase can be prevented if the sample is cooled during STM image acquisition. Therefore, the sample shown in Figure S3b was cooled with liquid nitrogen to about 110 K. The sample is not identical to that shown in Figure S3a, but was prepared in an identical process and with a similar final coverage of about 1.25 ML. We expect that about a quarter of the surface area is already covered with molecules in the second layer. Therefore, we interpret the lower-appearing, dark structures in the image as condensed molecules in the second layer. As expected, the darker areas cover roughly 25 % of the imaged area. Thus, we can conclude that a sample temperature of 110 K is sufficient to freeze out the diffusive motion of the molecules in the second layer.

While no molecular structure is visible in the bright and dark regions of the large area scan, only a slight change in the tunneling parameters reveals at least the existence of rows similar to the ones observed in Figure S3a. The defects imaged in the inset of Fig S3b do

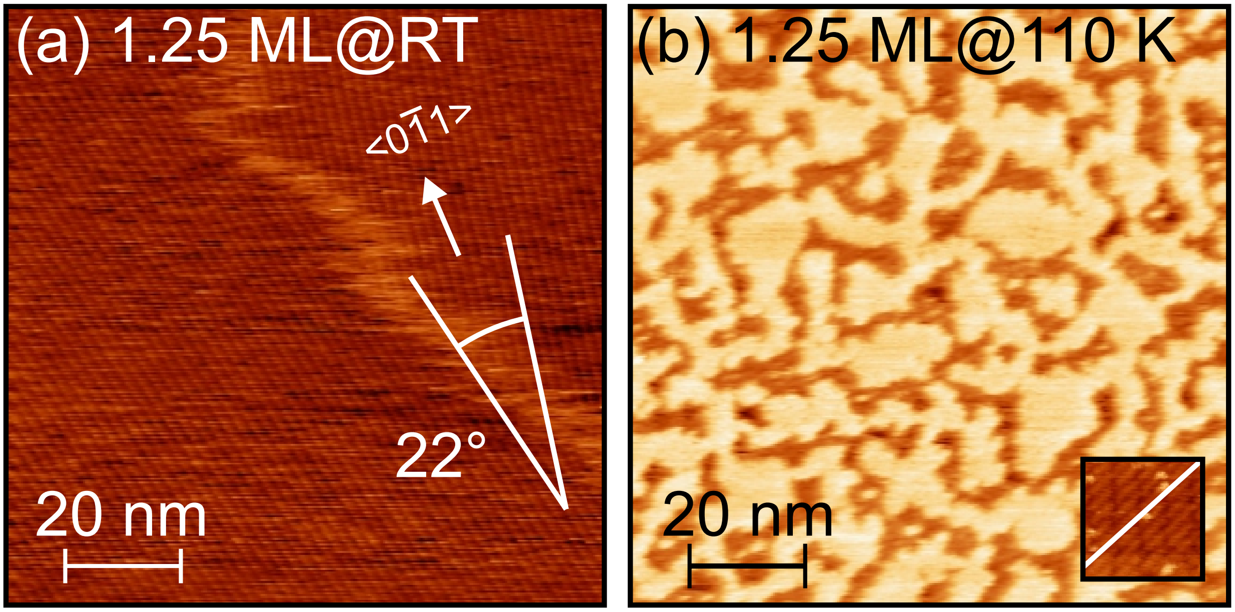

Figure S3: STM images of ≈1.25 ML (NapNC)AuCl deposited on a Au(111) surface. Both images show an area of $100 \times 100\,\mathrm{nm}^2$. (a) During imaging the sample was at room temperature. The STM parameters were $U_{\mathrm{sample}} = -1.0\,\mathrm{V}$ and $I_T = 7\,\mathrm{nA}$. A domain boundary, which appears particularly noisy, can be seen in the image. (b) The image was acquired at a sample temperature of about 110 K with $U_{\mathrm{sample}} = 700\,\mathrm{mV}$ and $I_T = 15\,\mathrm{nA}$. The inset shows a $20 \times 20\,\mathrm{nm}^2$ large area on the surface after changing the STM parameters to $U_{\mathrm{sample}} = 1.0\,\mathrm{mV}$ and $I_T = 25\,\mathrm{nA}$ and relocation of the tip. The solid white lines indicate the stacking direction of the dimers along the short axis of the unit cell.

not move. We can assume that the structure shown belongs to the first layer. For other parameters (not shown here), we were able to tunnel directly into the gold atoms of the substrate. We also observe that the same area shows an increasing defect density after repeated scanning with the STM tip. Due to such tip induced changes, the parameter range and time window allowing to resolve the dimer structure as in Figures 3a and 4a of the main article is rather limited.

3 Position Dependent LEED Images

Figure S4 shows a series of LEED images taken while manually (and therefore randomly) varying the position of the sample with respect to the impinging electron beam. The LEED patterns were recorded with an electron energy of 22.8 eV. To image the spots close to the central (0,0) reflex, the Au(111) single crystal was tilted slightly with respect to the LEED optics. The deposition of (NapNC)AuCl was stopped upon reaching a coverage Θ of about 1.2 ML: after a plateau, the transient of the mean electron yield decreases steeply here. Before the sample was examined in the LEED, it was annealed at 353 K for a total time of about 20 min. A comparison of Figure S4 with Figure 5a from the main article confirms that the surface is mainly covered with the molecular phase, which can be described by the following epitaxial matrix:

$$M_1 = \begin{pmatrix} 3.75 & 3 \\ 0 & 7 \end{pmatrix} \tag{S8}$$

Due to the symmetry of the substrate, we can assume that six equivalent domains contribute to the LEED image. When the position of the sample in front of the LEED optics was changed, certain groups of spot lose intensity while others gain intensity. These intensity variations were used to identify spots belonging to one of the six equivalent domains.

Since at some sample positions only one or two (instead of all six) orientations of the dense phase contribute to the diffraction pattern, it is tempting to assume that the sample

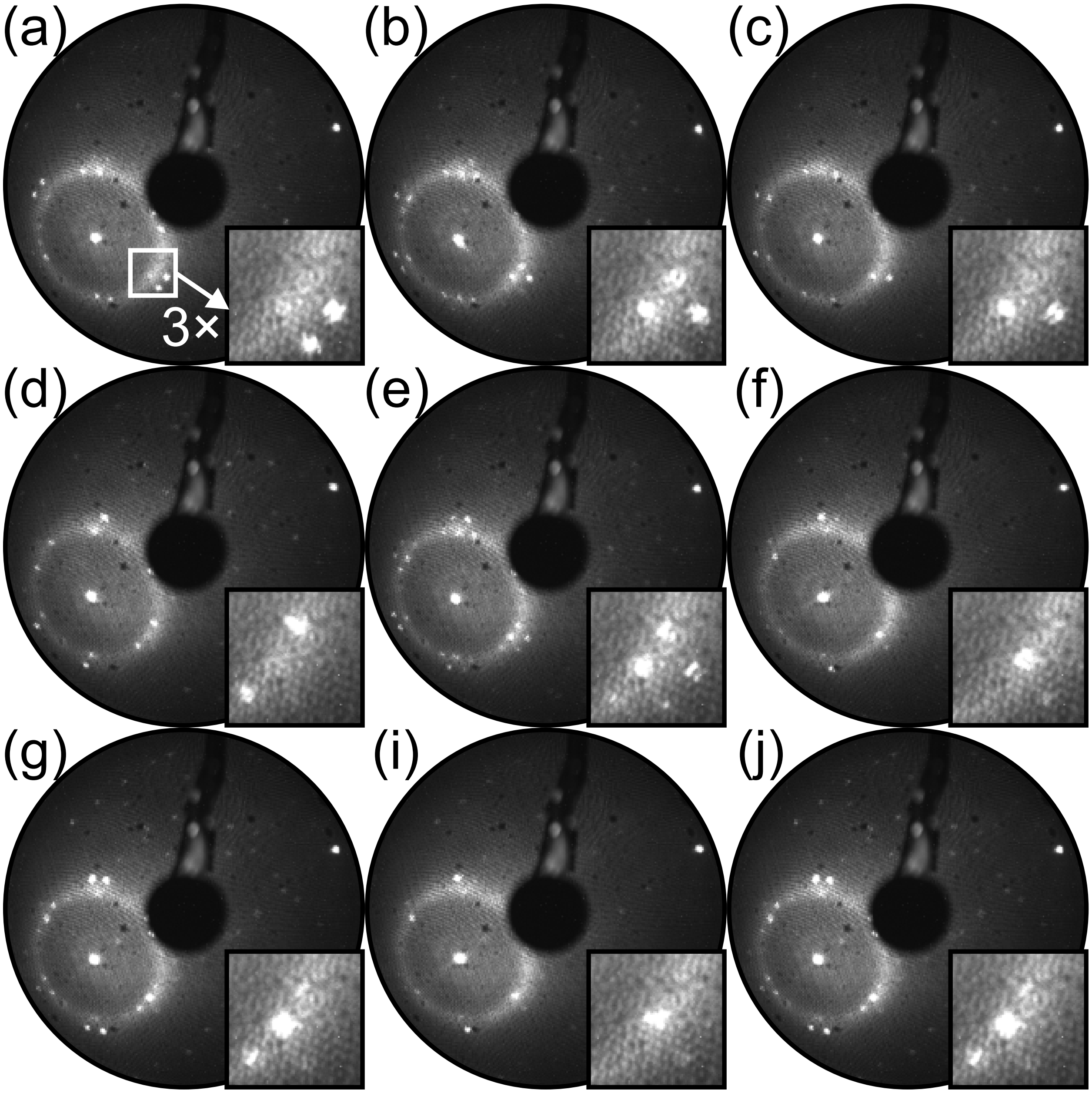

Figure S4: Series of LEED images taken while the Au(111) single crystal was randomly moved in front of the LEED optics. Therefore, different areas of the sample some 100 μm apart are imaged in panels (a–j). The (NapNC)AuCl coverage is $\Theta \approx 1.2\,\mathrm{ML}$. The sample was annealed for a total of 20 min at a temperature of 363 K. The energy of the electron beam was 28.2 eV.

area hit by the electron beam is covered with only one or two domains (see eq S8). This might indicate that extremely large domains formed during the annealing process, e.g. by Ostwald ripening.[7] Since terraces of the single-crystalline Au(111) substrate usually extend over a length of about 500 nm, such uniformly ordered domains should extend across many terrace boundaries. A more likely explanation could be that locally higher step densities of the substrate can trigger the molecules to preferentially arrange in a particular orientation with respect to these step edges.

A striking feature in all LEED images in Figure S4 is the bright halo around the (0,0) spot. The radius of the halo corresponds to the approximate size of the (NapNC)AuCl dimers. Since the total amount of molecules deposited fills more than a densely packed layer, it is likely that this halo corresponds the incoherent superposition of structure factors originating from the diffraction of individual dimers. These dimers form a dilute 2D gas phase in the second layer allowing for a certain rotational degree of freedom in the 2D plane.[8,9]

References

(1) Györök, M.; Wagner, T.; Gründlinger, P.; Monkowius, U.; Zeppenfeld, P. Aurophilic Molecules on Surfaces - Part I: (NapNC)AuCl on Au(110). *ACS Omega* **2023**, *8*, 30109–30117.

(2) Johnson, P. B.; Christy, R. W. Optical Constants of the Noble Metals. *Phys. Rev. B* **1972**, *6*, 4370–4379.

(3) Hobbollahi, E.; List, M.; Redhammer, G.; Zabel, M.; Monkowius, U. Structural and photophysical characterisation of gold(I) complexes bearing naphthyl chromophores. *Inorg. Chem. Commun.* **2016**, *65*, 24–27.

(4) You, Z. Z.; Hua, G. J. Refractive index, optical bandgap and oscillator parameters of organic films deposited by vacuum evaporation technique. *Vacuum* **2009**, *83*, 984–988.

(5) Azzam, R. M. A.; Bashara, N. M. *Ellipsometry and polarized light*; North-Holland Pub. Co., 1977; p 529.

(6) Polyanskiy, M. N. Refractive index database. `https://refractiveindex.info`.

(7) Bartelt, N. C.; Theis, W.; Tromp, R. M. Ostwald ripening of two-dimensional islands on Si(001). *Phys. Rev. B* **1996**, *54*, 11741–11751.

(8) Stadler, C.; Hansen, S.; Kröger, I.; Kumpf, C.; Umbach, E. Tuning intermolecular interaction in long-range-ordered submonolayer organic films. *Nat. Phys.* **2009**, *5*, 153–158.

(9) Kröger, I.; Stadtmüller, B.; Stadler, C.; Ziroff, J.; Kochler, M.; Stahl, A.; Pollinger, F.; Lee, T.-L.; Zegenhagen, J.; Reinert, F.; Kumpf, C. Submonolayer growth of copper-phthalocyanine on Ag(111). *New J. Phys.* **2010**, *12*, 083038.
